# Human Endogenous Retroviruses Are Ancient Acquired Elements Still Shaping Innate Immune Responses

**DOI:** 10.3389/fimmu.2018.02039

**Published:** 2018-09-10

**Authors:** Nicole Grandi, Enzo Tramontano

**Affiliations:** ^1^Laboratory of Molecular Virology, Department of Life and Environmental Sciences, University of Cagliari, Cagliari, Italy; ^2^Istituto di Ricerca Genetica e Biomedica, Consiglio Nazionale delle Ricerche, Cagliari, Italy

**Keywords:** HERV, endogenous retroviruses, innate immunity, interferon, evolution, autoimmunity, cancer

## Abstract

About 8% of our genome is composed of sequences with viral origin, namely human Endogenous Retroviruses (HERVs). HERVs are relics of ancient infections that affected the primates' germ line along the last 100 million of years, and became stable elements at the interface between self and foreign DNA. Intriguingly, HERV co-evolution with the host led to the domestication of activities previously devoted to the retrovirus life cycle, providing novel cellular functions. For example, selected HERV envelope proteins have been coopted for pregnancy-related purposes, and proviral Long Terminal Repeats participate in the transcriptional regulation of various cellular genes. Given the HERV persistence in the host genome and its basal expression in most healthy tissues, it is reasonable that human defenses should prevent HERV-mediated immune activation. Despite this, HERVs and their products (including RNA, cytosolic DNA, and proteins) are still able to modulate and be influenced by the host immune system, fascinatingly suggesting a central role in the evolution and functioning of the human innate immunity. Indeed, HERV sequences had been major contributors in shaping and expanding the interferon network, dispersing inducible genes that have been occasionally domesticated in various mammalian lineages. Also the HERV integration within or near to genes encoding for critical immune factors has been shown to influence their activity, or to be responsible for their polymorphic variation in the human population, such as in the case of an HERV-K(HML10) provirus in the major histocompatibility complex region. In addition, HERV expressed products have been shown to modulate innate immunity effectors, being therefore often related on the one side to inflammatory and autoimmune disorders, while on the other side to the control of excessive immune activation through their immunosuppressive properties. Finally, HERVs have been proposed to establish a protective effect against exogenous infections. The present review summarizes the involvement of HERVs and their products in innate immune responses, describing how their intricate interplay with the first line of human defenses can actively contribute either to the host protection or to his damage, implying a subtle balance between the persistence of HERV expression and the maintenance of a basal immune alert.

## Introduction

**Figure 1:**
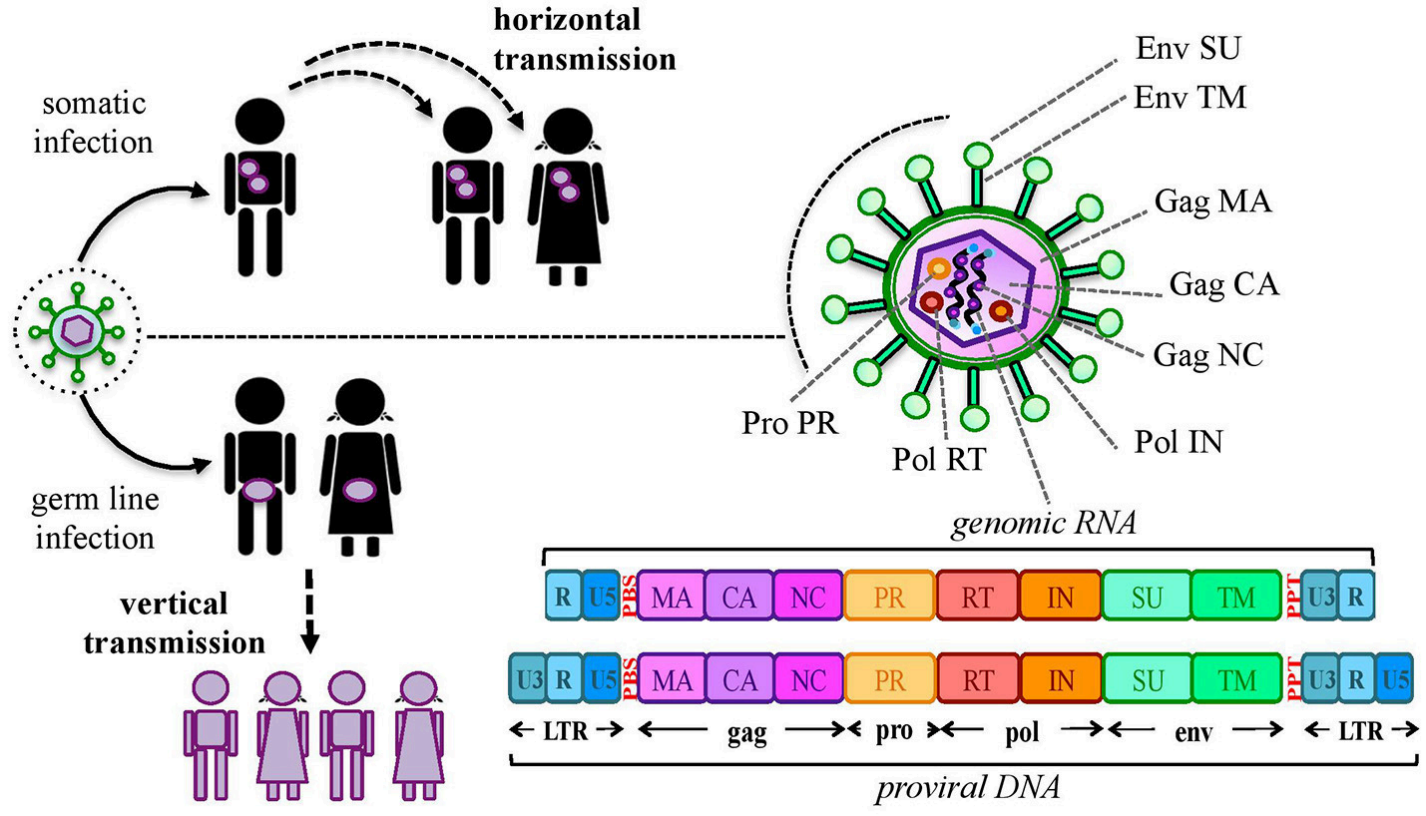
Origin and general structure of HERV sequences. Exogenous retroviruses normally infect a specific type of somatic cells, being diffused from a host to new individuals by a horizontal transmission. In the case of HERVs, the ancestral retroviral infection affected the germ line cells: in this way, the proviral sequences have been endogenized and vertically transmitted to all the cells of descendant individuals. HERVs have been so inherited in a Mendelian fashion to the offspring, being fixed in the human population. The general structure of a full-length HERV provirus is represented: the two Long Terminal Repeats (LTRs) are formed during the reverse transcription of the viral RNA genome and flank the *gag, pro, pol*, and *env* genes. The primer binding site (PBS) and the polypurine trait (PPT) are located between 5′LTR and *gag* and between *env* and 3′LTR, respectively. The viral genes encode for the structural and non-structural proteins found in the viral particle: *gag* matrix (MA), capsid (CA) and nucleocapsid (NC); *pro-pol* protease (PR)—reverse transcriptase (RT) and integrase (IN); *env* surface (SU) and transmembrane (TM) subunits. While in exogenous retroviral infections the integrated provirus is transcribed by the cellular machinery to release new virions, the HERV persistence within the host genome and the action of cellular editing systems led to the accumulation of mutations that often made the proviruses coding-defective and thus unable to produce infectious particles.

Our genome includes an impressive proportion of repetitive elements, among which human endogenous retroviruses (HERVs) account for about the 8% (1). HERVs are DNA sequences of retroviral origin that have been acquired along the last 100 million of years through multiple integrations by now-extinct exogenous retroviruses (2, 3). Peculiarly, while known retroviruses target the somatic cells, these ancestral infections affected the primate germ line, leading to the vertical transmission of HERV relics through the offspring (Figure 1). It is however still not clear if the HERV-originating exogenous retroviruses had germ line cells as main/unique target or infected this population by chance (4). In general, the mechanism that formed HERV insertions is analogous to the one used by exogenous retrovirus replication. In both cases, once into the cytoplasm, the RNA genome is reverse transcribed into a double-stranded DNA (dsDNA) by the viral reverse transcriptase (5). The so-obtained proviral DNA is then integrated in the host chromosomes through the viral integrase that interacts with cellular cofactors (6). At this point, the provirus expression generates a set of mRNAs encoding for the different viral proteins. In the presence of a functional reverse transcriptase, the full-length mRNA can also be reverse transcribed, producing a proviral cDNA theoretically competent for new integration events. The action of the host editing systems and the genomic substitution rate, however, often made HERV proviruses defective, leaving only a residual protein coding capacity but more often producing non-coding RNAs. HERVs share with exogenous retroviruses the typical proviral structure, being normally composed of two long terminal repeats (LTRs) that flank the internal portion of the viral genes *gag, pro*-*pol* and *env* (Figure 1). The LTRs are formed during the reverse transcription and have a regulatory significance for viral genes' expression, including promoters, enhancers and polyadenylation signals. The retroviral genes encode for the structural components, i.e. matrix, capsid and nucleocapsid (*gag*) and the envelope surface and transmembrane subunits (*env*), as well as for the enzymes involved in the viral life cycle, namely protease (*pro*), reverse transcriptase and integrase (*pol*) (Figure 1). While only these simple retroviral gene products have been identified in the majority of ERVs, some groups are known to have a more complex genome that encodes for additional proteins. This is the case of HERV-K(HML2) sequences, whose *env* gene—depending on the presence or the absence of a characteristics 292-bp deletion—can originate two splicing variants, Np9 and Rec, respectively (7, 8). Both proteins have been investigated due to their possible oncogenic properties [for a recent review, see (9)]. Only very recently, the presence of a putative *rec* gene has been also reported in another HERV-K group, namely HML10 (10). Aside from protein-coding genes, HERV proviruses harbor a primer-binding site (PBS) and a polypurine trait (PPT) located between the 5′LTR and *gag* and between *env* and the 3′LTR, respectively (Figure 1). Both have an important role during reverse transcription: the PBS binds the cellular tRNA priming the synthesis of the (–)strand DNA, while the PPT acts as a primer for the (+)strand DNA production.

The PBS sequence had also traditionally been used for HERV classification, and the current nomenclature still often relies on the tRNA type recognizing the different HERV groups' PBS (e.g., HERV-K for lysine tRNA, HERV-W for tryptophan tRNA, etc.). However, HERV classification is still ongoing, and the use of different methods in the last decades led often to multiple designations for the same HERV sequences and groups (11). Beside the use of the above PBS-based taxonomy, a minority of HERV groups has been named following unconventional criteria, such as the presence of a proximal cellular gene (e.g., HERV-ADP) or a peculiar amino acid motif (e.g., HERV-FRD). Clearly, such a lack of established rules generated confusion in the field, underlining the need of univocal guidelines for the naming of HERV groups, their single members and the latter expressed products (11, 12). Hence, the above mentioned nomenclature criteria are now considered inadequate, being not based on phylogenetic aspects and, regarding the PBS-based one, not taking into account the frequent occurrence of alternative PBS types (3, 13, 14). Currently, HERVs are broadly divided into three classes according to their similarity to exogenous members: class I (*gammaretrovirus*- and *epsilonretrovirus*-like), class II (*betaretrovirus*-like) and class III (*spumaretrovirus*-like). The classification of the various HERV groups is instead based on the phylogenetic relationships among the different groups - considering above all the highly-conserved *pol* gene - and can be corroborated by structural features found in all the members of the same genus or class (3, 11, 13). For what concerns the HERV nomenclature, a revised naming system has been introduced by the Human Genome Organization Gene Nomenclature Committee (12). Hence, for clarity, we provide a list of aliases in the presence of multiple HERV designation (Table S1).

An updated analysis of HERV sequences within the human genome has been recently performed (3) with the software RetroTector (15). A multi-step approach allowed to classify about 3200 HERV insertions in 39 “canonical” groups plus 31 “non-canonical” clades showing a mosaic structure arisen from recombination and secondary integrations (3). This classification provided a comprehensive overview of HERV diversity, being also a useful background for the still-ongoing characterization of the different HERV groups at the genomic level. Such an exact knowledge of the individual HERV sequences' localization and coding capacity could in fact represent a great advance in the understanding of their potential effects in both physiological and pathological contexts (16). In particular, while in the former some HERV locus-specific activities have already been demonstrated in placenta (9, 17–19), the tentative link between HERVs and human diseases has not lead to any definitive association, yet. In this regard, while many reports investigated the various HERV groups' overall expression in diseased tissues, very few studies tried to assign it to specific proviral loci of origin and to analyze their differential expression between patients and healthy individuals. This, together with the above-mentioned lack of knowledge about individual HERV sequences and the absence of standardized methodologies for HERV association studies, prevented until now the identification of precise molecular mechanisms of pathogenesis and, thus, the unambiguous link to any disorder (16, 20). Nevertheless, even if their significance in disease etiology is still uncertain, it is by now clear that HERVs can have an impact on the host in several ways. While some effects depend on the mere presence of HERV sequences within the human genome (16), in some other instances, HERV expression can provide RNAs and proteins potentially able to *trans*-regulate human genes and to influence the host immunity (21–24). It has been shown that HERV expression products can act as pathogen-associated molecular patterns (PAMPs), triggering the cellular receptors responsible for the first line of defenses (25–27). They can moreover provide antigenic epitopes recognized by lymphocytes (especially through molecular mimicry with exogenous viral molecules) and stimulating the onset of specific T- and B- cells (28–30). These mechanisms might be particularly relevant for the tentative involvement of HERVs in autoimmunity and inflammatory diseases. On the contrary, HERVs have also been involved in the downregulation of the host immunity, starting from their role in maternal immune tolerance to the fetus to their suggested protective action against excessive immune activation (31–34). The present review will discuss the interplay between HERV insertions and their expression products and our defenses, with a special attention to their contribution in shaping and influencing the human innate immunity.

## HERV role in the evolution and shaping of the human genome

About 75 years ago, the pioneer studies of Barbara McClintock suggested that transposable elements (TEs)—now known to constitute >40% of our DNA (1)—were not useless “junk DNA” but normal components of eukaryotic genomes that can have important regulatory roles (35). Nowadays, growing evidences confirm that TEs had a crucial role in the shaping and evolution of vertebrates' genomes, contributing to the establishment of lineage-specific patterns of gene expression (36, 37). TE insertion appear to be highly conserved among mammals, showing an increased density in the proximity of cellular genes and being a main source of transcription factor binding sites and regulatory signals (37–42). This points out their relevance for the human development and transcriptional modulation throughout evolution (36, 38). Of course, the majority of TEs have been acquired several million of years ago, being by now silenced due to the accumulation of mutations in their coding sequence and to various cellular mechanism of transcriptional repression, such as histone hypermethylation. Despite this, many TEs are likely involved in a long-term co-evolution with the host, that exerted firstly a selective pressure against their detrimental effects and led then in some instances to their cooptation for biological processes (43, 44). Intriguingly, even the general epigenetic silencing of TEs could represent an ancestral adaptation of the harboring organism that, initially, evolved defense systems to downregulate them and, then, developed strategies to control gene expression through their exaptation (44). In this view, the sporadic loss of epigenetic regulation could be not only linked to certain developmental stages and disorders, but might also constitute an opportunity to boost evolvability (44). Accordingly, despite controlling mechanisms, many HERV sequences still retain a residual expression capacity, leading to the production of either coding or non-coding RNA transcripts that can both influence the host biology. One of the most remarkable examples of the HERV impact on vertebrate physiology is represented by “syncytins,” an ensemble of Env proteins encoded by different HERV sequences in all eutherian mammals through a process of convergent evolution (45). Syncytins have in fact been domesticated independently by the various species, providing common and important functions for placenta development and physiology. In the case of the human genome, two *env* loci, namely *ERVWE1* (HERV-W, 7q21.2) and *ERVWE2* (HERV-FRD, 6p24.1), encode for the coopted Env proteins syncytin-1 and -2, respectively (17, 18). While syncytin-1 has a pivotal role in placental syncytiotrophoblast development and homeostasis (17, 18, 46, 47), syncytin-2 is thought to be involved in the maternal immune tolerance to the fetal allograft (32). The role of these and other HERV-derived Env proteins have been recently reviewed elsewhere (9).

**Figure 2:**
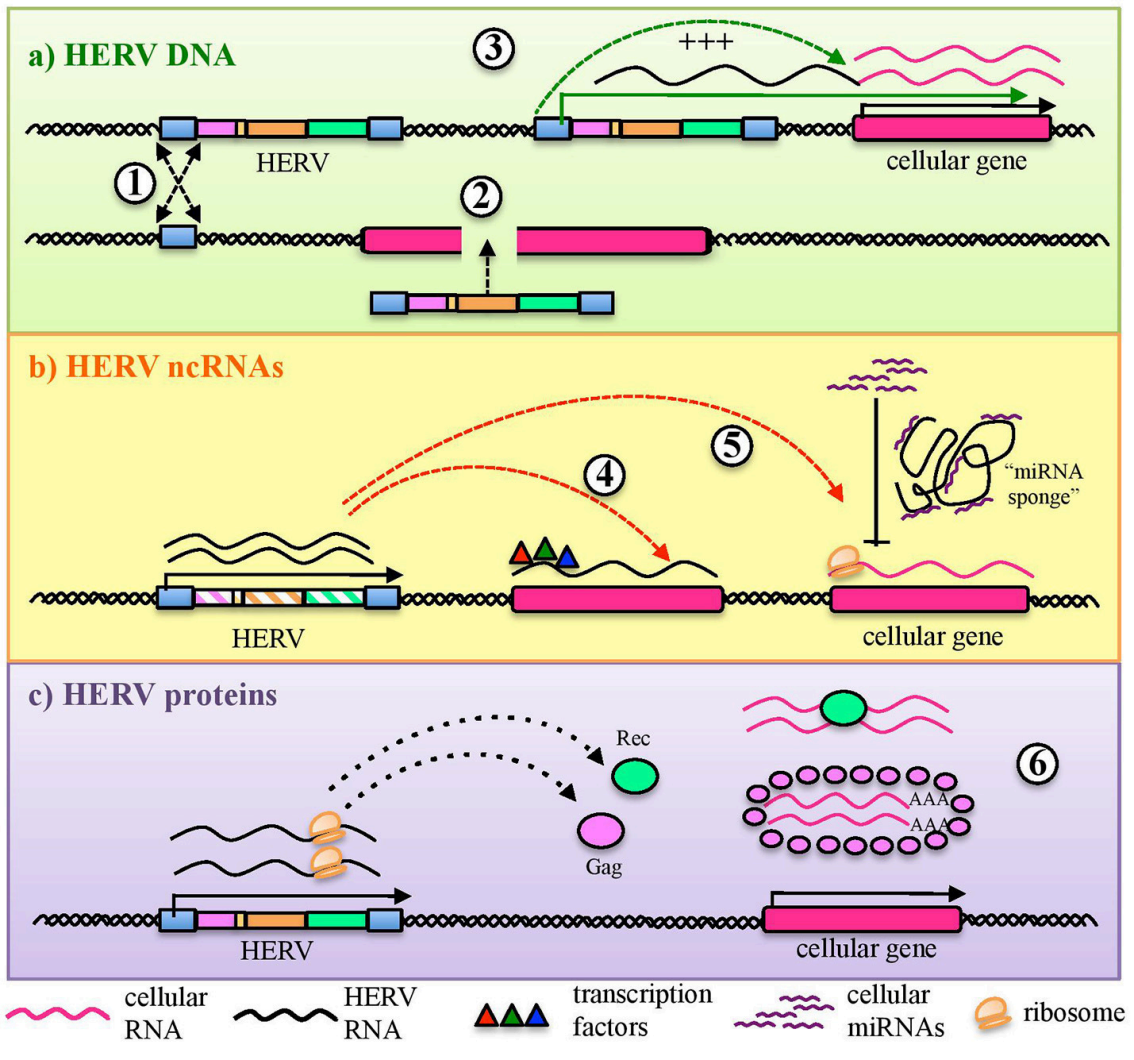
HERV role in the regulation and shaping of human gene expression. HERVs can influence the host gene expression at several levels. **(a)** Integrated DNA sequences can trigger chromosomal rearrangements by non-allelic homologous recombination (1) or disrupt co-localized genes through insertional mutagenesis (2). Moreover, HERV sequences integrated in proximity to a cellular gene can provide alternative promoters or enhance its expression through LTR *cis*-regulatory elements (3). **(b)** HERV non-coding RNAs (ncRNAs) can also be able to *cis*-regulate cellular genes, even through the recruitment of cellular regulators (e.g.: transcription and splicing factors) (4). In addition, HERV ncRNA have been reported to act as “microRNA sponges,” binding and dampening microRNA families responsible for post-transcriptional modifications (5). **(c)** Finally, some HERV proteins can also regulate genic expression through their interaction with cellular mRNAs and the modulation of their transfer and ribosome occupancy (6).

Aside from protein production, the thousands of HERV sequences dispersed in our DNA contributed to the evolution of the primates' genome by providing an abundant source of regulatory elements. It is well known that our genetic information is organized in regulatory networks, involving both *cis*- regulatory sequences and *trans*-acting genes, and that their interaction is at the base of cellular plasticity and evolution (23, 44). Also HERVs participate to this complex interplay, being able to regulate the host genes' activity in several ways and at different expression levels (Figure 2). In fact, even the sole presence of HERV proviruses and solitary LTRs can influence cellular genes' activity (Figure 2a). Repetitive elements' insertion is a source of genomic modifications, possibly leading to the disruption or insertional mutagenesis of co-localized genes or promoting chromosomal rearrangements through non-allelic homologous recombination (Figure 2a). This was reported in male infertility, in which the intra-chromosomal recombination between two homologous HERV-I sequences located on chromosome Y is responsible for the microdeletion of the azoospermia factor a (48). Moreover, HERV LTRs can provide *cis*-regulatory activity to nearby cellular genes by enhancing their transcription or even providing alternative promoters and splicing signals, also with a remarkable tissue-specificity (Figure 2a). A representative example is the HERV-E provirus integrated upstream of an ancestral amylase gene and acting as a specific enhancer in parotid glands (49, 50). Furthermore, despite the frequent loss of protein-coding capacity, also the abundant production of HERV-derived non-coding RNAs (ncRNAs) such as microRNA and long ncRNA, may likely provide *cis*-regulatory elements able to modulate the expression of the host genes, either alone (e.g., providing a recognition motif for an RNA-binding protein) or in concert with cellular transcription factors (Figure 2b). This occurs for example in human embryonic stem cells, whose pluripotency depends on nuclear long ncRNAs expressed by a HERV-H element and recruiting specific cellular transcriptional activators (51). In addition, HERV ncRNAs can act as “RNA sponges” binding and dampening microRNA families involved in the post-transcriptional regulation of gene expression, given that several human microRNAs have high sequence homology with HERVs (52–54). Such a miRNA sponge activity has been reported in the positive regulation of pluripotency in embryonic stem cells, which depends on the interaction of a HERV-H long ncRNA (HPAT5, locus 6q27) to complementary sequences in the let-7 microRNA family (54, 55) (Figure 2b). Finally, if a HERV protein is produced, it can be able to modulate the host genic expression through the biological activities previously involved in the virus life cycle and now providing new cellular functions (21) (Figure 2c). As an example, HERV Gag and Rec proteins are known to influence the stability, localization and translation of cellular transcripts (21). Accordingly, HML2 Rec was reported to interact with ~1,600 cellular mRNAs in embryonic cells and, especially, to influence their ribosome occupancy, possibly suggesting regulatory function coopted for early development (56). Similarly, the Arc Gag-like protein derived from an ancient Ty3/gypsy retrotransposon has been repurposed during brain evolution to mediate communications between neural cells, having an important role in the development and plasticity of the nervous system (57–59). In particular, Arc has been shown to assemble into capsids that include mRNA sequences to be transferred from a neuron to new recipient cells through extracellular vesicles, then undergoing activity-dependent translation (60).

Beside the role of individual HERV sequences, it has been proposed that the ensemble of TEs widespread in the human DNA can have a more extensive role. By constituting a sort of parallel regulatory network, TEs can possibly influence multiple host genes and, thus, shape whole pathways involved in complex cellular processes (22, 36, 61). Accordingly, more than one third of p53 binding sites in the human genome have been dispersed by class I HERV sequences, which have become major components of the p53 regulatory network (62). Hence, the acquisition of HERV insertions could have acted as a driving force for human genome plasticity and cellular networking, and, as described below, such TE-dependent modeling and functional renewal has been particularly critical in the evolution of innate immunity. Vertebrate genomes have been subjected to substantial rearrangements through the acquisition of HERVs and, intriguingly, such colonization has been concomitant to the development of important immune pathways (24). Major shifts have occurred in both innate and adaptive immune systems, increasing the complexity and specificity of vertebrate antiviral defenses (24). The loci of primate MHC (major histocompatibility complex) are in fact characterized by a high density of HERV integrations, which contributed to their remarkable plasticity (63) and led to genic variations among species. As an example, a HERV-K(HML10) provirus inserted within the ninth intron of human complement *C4A* gene (MHC class III), also called HERVKC4, is responsible for its dichotomous size variation (10, 64–66). Besides generating physical changes among species, such TE insertions in immune gene introns could also account for regulatory effects, being present mostly in antisense orientation and subjected to bidirectional transcription (10). Noteworthy, mRNA sequences originated by classes of genes with a relatively recent expansion, such as the ones involved in immunity, are enriched in TEs, which are not present in the transcripts arising from highly conserved genes with basic functions (38). Overall, one of the soundest evidences about the role of HERVs in the shaping of pivotal immune systems regards the interferon (IFN) network, a crucial antiviral pathway for innate immunity and a fundamental effector to initiate and maintain adaptive responses (67). Intriguingly, Chuong and coauthors showed that HERV insertions greatly contributed to the evolution and amplification of IFN transcriptional network, dispersing independently a wide number of IFN-inducible enhancers in many mammalian genomes (22). In particular, the experimental deletion of a subset of these endogenous elements in human cell lines affected the activity of neighboring genes induced by IFN, impairing thus important immune pathway such as the AIM2 inflammasome (22). Furthermore, due to their residual regulatory activities, HERV LTRs can act as promoter and/or enhancers after IFN-mediated stimulation (68). This has been reported for HERV-K(HML2) LTRs, which harbor two IFN-stimulated response elements (ISREs) activated by the IFN signaling, leading to the increased HML2 expression in response to inflammation (69, 70). In line with this, HERV-K *env* genes transcription can be stimulated by IFN α, encoding Env superantigens responsible for polyclonal T-cell activation (71).

## HERVs as activators of antiviral innate immunity

Innate immunity is the first and most ancient line of defenses against microbial infections, acting by a complex network that is conserved throughout the animal kingdom (72). When an infectious agent overcomes the organism physical barriers, the presence of conserved PAMPs (i.e., lipids, proteins, glycans, and nucleic acids) allows their prompt recognition by innate immunity sensors, namely pattern recognition receptors (PRRs). PRRs are germ line-encoded receptors with a pivotal role in antiviral defenses, as well as in the response to self-injuries (73), recognizing PAMPs and danger-associated molecular patterns (DAMPs), i.e., molecules present in damaged or stressed tissues (74).

In vertebrates, PRRs include five major classes of receptors that are localized either transmembrane or in the cytosol (tm- and cytPRRs, respectively) of different cell types (73). tmPRRs are represented by the Toll Like Receptors (TLRs), detecting PAMPs either on the cell surface or in the endosomal compartments. cytPRRs include RIG-I-like receptors (RLRs), NOD-like receptors (NLRs), C-type lectin receptors (CLRs) and DNA sensors, all recognizing intracellular PAMPs (26, 75–77). cytPRRs are usually present in cells that can be actively infected by a given class of microbial agents, while tmPRR cell-extrinsic recognition is independent from the cell infection and usually occurs in cells devoted to pathogen detection (78). In both cases, PRR stimulation by microbial PAMPs activates a complex cascade of signaling that triggers the production of various pro-inflammatory molecules, including cytokines, chemokines and type I IFN (78, 79). These effectors have a double role. On the one side, they quickly establish an antimicrobial environment to counteract the infection. On the other side, the activation of PRRs expressed on antigen-presenting cells (APCs), especially dendritic cells (DCs), evokes the development of a long-lasting adaptive immunity, leading to the onset of specific cellular and humoral defenses (78). The latters are mainly represented by the clonal expansion of naïve cytotoxic T cells and the production of antibodies by B lymphocytes. Other important APCs are macrophages and B lymphocytes, which are albeit mostly involved in the stimulation of already-activated T cells. The overall innate immune signaling has been reviewed elsewhere (72, 79–81): in the following sections we will focus our attention on the specific pathways relevant to exogenous retrovirus sensing and, thus, possibly involved also in HERV immune recognition.

**Figure 3:**
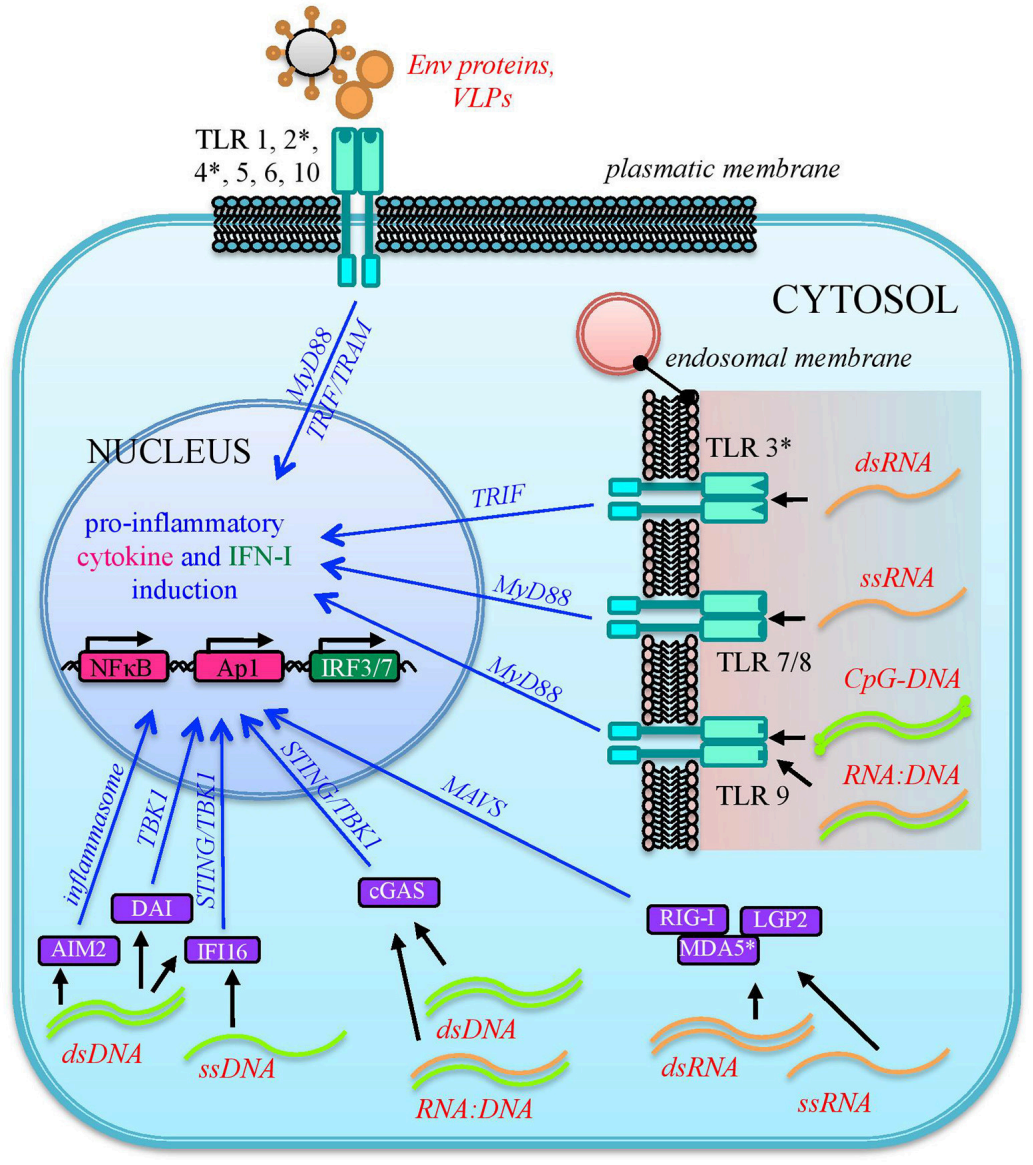
Sensing of HERV molecules by innate immunity PRRs. Different HERV proteins and nucleic acids (overall indicated in red) can theoretically be detected as PAMPs or DAMPs by cellular sensors localized at the plasmatic or endosomal membranes (transmembrane PRRs or Toll Like Receptors, cyano) or present as soluble factors in the cytosol (cytosolic PRRs, violet). The sensing of these viral molecules by both kind of PRRs triggers a signaling cascade (blue arrows) that leads to the nuclear activation of immune genes encoding for pro-inflammatory effectors, represented by cytokines and type I IFN. The individual PRRs for which a direct interaction with HERV molecules has been reported are marked with an asterisk.

HERVs are integral parts of the human genome since millions of years, are highly transcribed during embryonic development and expressed at variable levels in several adult tissues. Hence, from the immunological point of view, HERV expressed products somehow stay at the interface between self-molecules and microbial antigens. In fact, also depending on the cellular and immunological surrounding, HERV-derived molecules can be either tolerated by human defenses or able to stimulate the human immunity, leading to their intensive investigation in various autoimmune diseases. In addition, the presence of other immune stimuli has been shown to be capable to influence HERV expression, suggesting a complex and multifaceted interplay that is still not completely clarified. In principle, the innate immune pathways activated by HERV-derived products are the same involved in the first line antiviral defenses counteracting exogenous retroviruses, including both tm- and cytPRRs (26) (Figure 3). In humans, TLRs from 1 to 10 are inserted in plasmatic or endosomal membranes and expressed on both innate immune cells and non-immune cell types, while cyt-PRRs are soluble elements normally found in the cytosol or migrating to it when stimulated by the presence of retroviral molecules following cellular infection (80, 82). Both tm- and cytPRRs can potentially recognize HERV nucleic acids and proteins either due to their molecular identity with exogenous viral PAMPs or as DAMPs (26, 74). The upregulation of HERV transcription, even in the absence of a causal role in the initiation of immune-related pathogenesis, can thus provide numerous HERV-derived PAMPs or DAMPs able to further prompt inflammation, contributing to the symptomatology. This could likely occur in diseases such as autoimmunity and cancer, which have in common a general epigenetic de-regulation known to strongly liberate the non-specific retroelements' expression (9, 16). Hence, even if no definitive associations have been described yet, a growing body of studies is confirming that HERVs may have an impact on the human immune homeostasis, starting form their interaction with PRRs known to have a central role in the early response to HIV-1 infection, i.e., TLRs, RLRs, and cytoplasmic DNA-sensors (83).

### Sensing of HERVs by transmembrane PRRs

As mentioned above, different tmPRRs can potentially be activated by either HERV nucleic acids or proteins (84) (Figure 3). TLRs are the first PRRs to be identified and have been studied in greater detail: they can sense viral molecules either on the cellular surface, through plasmatic membrane TLRs (1, 2, 4, 5, 6, and 10), or in the endosome, through TLRs localized in the endosomal membranes (3, 7, 8, 9) (80). All TLRs have a common structure, characterized by a first domain that binds specific ligands, protruding outside the cell surface or inside the endosome, and a second conserved cytosolic domain that is required for the intracellular signaling. Their activation relies albeit on different types of molecules. Plasmatic membrane TLRs can recognize retroviral proteins, either as individual molecules or as components of viral-like structures (Figure 3). Accordingly, TLR2 and TLR4 are both able to detect the Env proteins of retroviruses, including HIV-1 gp120 that induce NF-κB activation and proinflammatory cytokine secretion (85). In line with this, both receptors are upregulated in DCs, macrophages and peripheral blood mononuclear cells during HIV infection (86–88). Endosomal TLRs can instead be involved in the sensing of different retroviral nucleic acids, which should however be auto-phagocytized from the cytoplasm to the endosomal lumen to be detected (26, 74) (Figure 3). In particular, one of the most immunogenic nucleic acids PAMPs is dsRNA, which is normally not found in uninfected cells. Viral dsRNA can stimulate an innate response through TLR3 signaling (72) and, interestingly, the immune activity elicited by TLR3 agonists has been shown to provide protection from different viral infections, among which certain HIV strains (89). TLR3 has been also implicated in the HIV-1 transactivation (90), replication (91), and inflammation (92). Following proviral transcription, HERV ssRNA could be recognized by both TLR7 and -8. The same TLRs, in fact, are important for the recognition of the HIV RNA genome during acute infection, probably through the recognition of guanosine- and uridine- rich ssRNA that stimulates the production of IFN-α and proinflammatory cytokines by DCs and macrophages (93). In the presence of reverse transcriptase activity, the HERV ssRNA can serve as a template to the synthesis of RNA:DNA hybrids, which can be sensed by TLR9 (94). The latter was in fact shown to sense RNA:DNA hybrids presenting viral-derived sequences, leading to the secretion of IFN-I and pro-inflammatory cytokines in DCs (94). TLR9 is also the sole DNA-sensing TLR and specifically binds unmethylated CpG-rich DNA (72, 82) that, however, is present mostly in bacteria and DNA viruses (72, 80). Recently, TLR9 agonists have been reported to induce HIV production in latently infected cells, being investigated as possible therapeutic strategies to attack the proviral reservoir (95, 96).

In any of the above pathways, once activated, tmPRR dimeric receptors prime a complex intracellular cascade of signaling that, through several kinases and ubiquitinases, could lead to the activation and nuclear translocation of transcription factors that stimulate the expression of cytokines, chemokines and type I IFN (IFN-I) establishing an initial antiviral status and stimulating the adaptive immune defenses. Currently, even if all the mentioned tmPRRs could be potentially able to sense HERV-derived molecules, there are yet limited findings reporting the direct interaction between these ligands and few plasmatic membrane TLRs. Contrarily, endosomal TLRs were widely shown to be involved in the control of murine ERVs (97) albeit still remaining poorly characterized for their interplay with human TEs.

In one of the most investigated groups, the HERV-W, the Env surface subunit has been characterized to be a potent stimulator of TLR4, harboring remarkable pro-inflammatory properties that might contribute to multiple sclerosis immunopathogenesis (98–100) (Table 1). The HERV-W Env was shown to interact with both TLR4 and CD14, inducing proinflammatory molecules that are prevalent in multiple sclerosis, such as IL-1, IL-6 and tumor necrosis factor α [(99, 109, 110), Table 1)]. These immune effectors can likely contribute to the damage of brain populations, including DCs, oligodendrocytes and astrocytes (111). HERV-W Env was in fact shown to activate DCs and promote a Th1-like immune response (99), adding mechanistic insights to the tentative link between HERV-W sequences and multiple sclerosis [recently reviewed in Grandi and Tramontano (16)]. The interaction between HERV-W Env and TLR4 was also investigated in both murine and human oligodendrocyte precursors, leading to an increased production of cytokines and inducible nitric oxide synthase (101) (Table 1). In line with this, HERV-W Env overexpression in mice led to the development of experimental allergic encephalomyelitis (112) and inflammatory hallmarks typical of myelin injuries (113, 114). Such a HERV-W Env mediated pro-inflammatory effect was responsible for reduced oligodendrocytes differentiation and affected myelin expression and renewal (101), as also demonstrated by the fact that treatment with a specific antibody for HERV-W Env (GNbAC1) was able to rescue myelin expression (115). In addition, HERV-W Env hold a significant superantigen activity (116), that has also been proposed to play a role in demyelination, by evoking a polyclonal non-specific T-cell activation and the massive release of multiple cytokines (98, 109). A similar interaction between HERV-W Env and the TLR4 of pancreatic β cells has been proposed to promote autoimmune reactions and to affect insulin secretion in type I diabetes patients (117).

**Table 1:** HERVs for which a direct role in the modulation of innate immunity has been reported.

**Group**	**Locus**	**Name**	**Type**	**Effect**	**Mechanism**	**Reference**
HERV-W	?	–	Env SU	immune-activation	stimulation of CD14 and TLR4, monocyte and dendritic cell activation, inflammatory cytokines production	(99)
	?	–	Env	immune-activation	stimulation of cytokine and inducible nitric oxide synthase production	(101)
	HERVWE1	syncytin-1	Env TM	immune-suppression	Th1 to Th2 shift in maternal immune-tolerance	(102)
HERV-FRD	HERVWE2	syncytin-2	Env TM	immune-suppression	Th1 to Th2 shift in maternal immune-tolerance	(32)
HERV-H	HERV-H3	Env-59	Env TM	immune-suppression	inhibition of inflammation effectors	(103, 104)
	HERV-H17	Env-60	Env TM ISD	immune-suppression	tumor immune escape by stimulation of CCL19 chemokine and expansion of pluripotent CD271^+^ cells	(105)
HERVP71	?	HERVP71A	LTR	immune-suppression	enhancer for HLA-G in human extravillous trophoblasts, inhibiting natural killer cytotoxicity	(106)
HERV-K(HML2)	HERV-K108	–	Env TM	immune-suppression	inhibition of T cells activation, cytokine release and immune gene expression	(107)
	6p21.33 (PSORS1 gene)	–	Pro dUTPase	immune-activation	stimulation of TLR2, induction of NF-κB and Th1+Th17 cytokines	(108)

A second evidence of HERV pro-inflammatory potential comes from the investigation of HERV-K(HML2) group expression in psoriasis, another poorly understood autoimmune disorder. In this context, wild type and mutated HML2 dUTPases (metallo-enzymes that hydrolyzes dUTP preventing its incorporation into the viral DNA) were shown to interact *ex vivo* with TLR2 (108). Such interaction stimulated the expression of NF-κB and induced Th1 and Th17 cytokine production in DCs and Langerhans-like cells as well as, even if at lower levels, in keratinocytes (108) (Table 1). This could suggest a possible role of HML2 dUTPase in the immunopathogenesis of psoriatic lesions, even if these observations have been performed in primary cells from healthy individuals and not confirmed *in vivo*. A subsequent study explored the HML2 dUTPase locus in a large number of psoriatic patients, reporting the association of some single nucleotides variants with a lower susceptibility to the disease (118). A small subset of these patients was also tested for dUTPase-induced cellular immunity, showing frequently increased B- and T-cell responses that were albeit not further characterized at the molecular level (118).

Finally, the stimulation of endosomal TLR3 by HERV dsRNA molecules has been observed after the treatment with DNA methyltransferase inhibitors, anticancer agents that were shown to remove methylation from the HERV promoter regions, causing their reactivation and subsequently triggering IFNα and β responses (27). Interestingly, such a HERV-mediated immune-stimulation is probably at the base of demethylating agent anticancer effect (see below).

### Sensing of HERVs by cytosolic PRRs

Besides transmembrane TLRs, other PRRs devoted to the antiviral response are present in the cytosol as soluble factors or reach this compartment after the sensing of microbial nucleic acids. Among them, the most relevant for antiviral immunity include the RLRs retinoic acid inducible gene I (RIG-I), sensing short dsRNA and ssRNA with a 5′-triphosphate moiety, and MDA5 (melanoma differentiated associated gene 5), that detects long dsRNA molecules (72). RIG-I was shown to be a crucial intracellular sensor for HIV nucleic acids, recognizing complex secondary structures in HIV RNA and stimulating IFN-I release by mononuclear cells (83). Accordingly, as a viral immune escape mechanism, HIV protease was shown to target RIG-I for lysosome degradation (119). Contrarily, MDA5 was not affected by HIV protease-mediated degradation (119), even if its upregulation has also been observed in HIV infection (120). Other cytPRRs, namely DNA sensors, recognize instead DNA of viral origin that, differently from nuclear DNA, can be found in the cytoplasm during retroviral replication. Particularly, in HIV-1 infected cells, the replication intermediates cDNA, ssDNA, and RNA:DNA hybrids can all be detected by cytoplasmic DNA sensing proteins (83, 121). Viral dsDNA can be sensed by the DNA-dependent activator of IFN (DAI) and the IFNγ inducible protein 16 (IFI16), both triggering the STING-TBK1-IRF3 signaling, as well as by the absent in melanoma protein 2 (AIM2), activating the inflammasome pathway (26, 82, 122). Another important sensor for cytoplasmic dsDNA is cGAS (cyclic GMP-AMP synthase), which can also recognize DNA-RNA hybrids and mtDNA released after mitochondrial damage (82). The main dsDNA sensors relevant to HIV infection are IFI16 and cGAS, which are both upregulated in the absence of antiretroviral therapy and associated with chronic immune activation (123). For what concerns foreign ssDNA, stem-rich secondary structures in HIV-1 ssDNA were shown to induce IFI16 in macrophages (124).

Given that retrovirus replication takes place mostly in the cytoplasm, cytPRRs are known to be crucial to the detection of RNA and DNA originated by exogenous retroviruses, while very few evidences about the molecular sensing of HERV nucleic acids are available yet (26). Particularly, similarly to TLR3, also MDA5 has been shown to sense HERV dsRNA triggered by the treatment with DNA methyltransferase inhibitors, evoking IFN production (27). A role in the prevention of HERV DNA/RNA accumulation has been proposed for certain enzymes involved in the cytoplasmic homeostasis of nucleic acids, which could possible provide some protection against HERV-mediated immune activation (9). Such hypothesis derives from observations made in mice deficient for the 3′ → 5′ exonuclease 1 (Trex1), in which the accumulation of endogenous retroelements' cDNA led to the activation of innate DNA sensors and the production of IFN (125–127). Even if a similar function is still to be confirmed for human Trex1, this enzyme was shown to restrict the reverse-transcribed DNA from endogenous retroelements which instead accumulated in Trex-1 deficient cells (125). Moreover, patients with Aicardi-Goutières autoimmune syndrome lack this enzyme and show inflammatory and IFN-I responses that could possibly be sustained by HERV nucleic acids accumulation. Hence, Trex1 could have a role in the control of HERV cDNA-mediated immune activation (125, 128), also considering that this cellular protein was recently reported to prevent cGAS or IFI16-mediated recognition of HIV-1 replication intermediates, being possibly involved in viral immune escape mechanisms (129). Similarly, the 3′ → 5′ RNA exoribonuclease SKIV2L has been recently involved in the exosomal degradation of endogenous RNA molecules in the cytosol, thus possibly preventing also HERV RNA accumulation that could trigger viral RNA-sensing receptors (130).

### Consequences of innate immunity stimulation

Independently from the cytosolic or membrane-associated localization, the sensing of viral molecules by innate immune receptors evokes the production of pro-inflammatory effectors (such as IFN, cytokines and chemokines), which promptly establish an antiviral status. This immune activation contributes to contain the infection and prepares the ground for subsequent adaptive immune responses, mediated by T- and B-lymphocytes that elicit specific cellular and humoral defenses, respectively. Overall, such innate-adaptive signaling is fundamental to counteract exogenous viruses, and the resulting immune activation is generally able to eliminate the infectious triggers and then to shut down. In the case of HERV molecules, however, their stable presence and expression in the organism could provide continuous triggers to the host immune sensors. In fact, the main findings linking HERVs to autoimmune and inflammatory disorders rely on the chronic stimulus exerted by endogenous retroviral molecules, which could sustain molecular mimicry events based on the sequence identity between HERV products and either exogenous viruses or body components (16, 28, 30, 131, 132). Worth to note, after the initial immune activation, the production of IFN establishes a positive-feedback that further upregulates IFN-stimulated genes by both a paracrine and an autocrine loop, fostering chronic inflammation and autoimmunity. The same antiviral status prompted by HERV expression can hence create a vicious circle in which inflammatory molecules and epigenetic dysregulation further upregulate HERV expression (26, 69, 84). For all these reasons, even if no definitive etiological evidences have been reached yet, the role of HERVs in triggering innate defenses could contribute to autoimmune pathological developments and, hence, be a valuable therapeutic target. Accordingly, monoclonal antibodies against HERV-W Env proteins are currently under clinical trials as innovative approach against multiple sclerosis (133, 134) and type I diabetes (117). In addition, even in the absence of a direct immunogenic role of HERV-derived proteins, the presence and nucleotide variability of HERV loci in proximity to key immune genes should be taken into account for their possible influence on the latters, especially in the context of genome wide association studies. These analyses are by now an established method to identify risk loci linked to autoimmune conditions, but rarely take into account HERV sequences and their networking with cellular genes. As an example, MS-associated SNPs showed a significant enrichment of HERV insertion and potential HERV ORFs in their genetic neighborhood as compared to control SNPs (135, 136). Accordingly, SNPs in the antiviral gene TRIM5 were shown to be negatively correlated to MS, while SNPs around a HERV-Fc1 locus on chromosome X had a significant association with the disease, with a risk that was strongly influenced by the two genes in an additive fashion (137). Hence, besides protein production, the genetic analysis of HERV loci in the neighborhood of immune genes, especially if presenting key immune roles, can provide insights on inter-individual variants concurring to autoimmunity risks.

## HERVs as inhibitors of innate immunity

In parallel to pro-inflammatory effects, HERV-derived peptides have also been implicated in immune-suppressive mechanisms. The latter mainly involve Env transmembrane subunits, which hold a characteristic immunosuppressive domain (ISD) conserved among retroviral Env proteins. In animal exogenous retroviruses, the ISD counteracts the host antiviral responses (138, 139) while, in the case of HERVs, such element has been occasionally coopted for the maternal immune tolerance during pregnancy (32, 45). In this physiological status, a subtle immune balance must allow the invasion of fetal trophoblasts, which present also paternal antigens, albeit maintaining the ability to protect the organism from microbial infections. Pregnancy is hence accompanied by the repression of cellular immunity through the shift from Th1 inflammatory cytokine production (TNF-α, IFN-γ, and IL-2) toward an anti-inflammatory Th2 cytokines response (IL-4, IL-5, IL-10), to prevent cytotoxic processes potentially harmful to the fetus (140, 141, 102). Such shift is thought to be mainly due to syncytin-2 ISD, which is highly preserved and shows strong immunosuppressive potential (32). However, also syncytin-1 was shown to be able to inhibit cytokine production in blood, suggesting a possible contribution in the maternal immune shift (102).

Besides these domesticated proteins, other HERV Envs have shown immunosuppressive activity. The transmembrane subunit of a HERV-K(HML2) sequence was reported to inhibit T cells activation in a similar way to the HIV ISD, influencing cytokine release and immune gene expression (107). Similarly, a HERV-H Env protein (Env-59) ISD showed anti-inflammatory potential in a mouse model of arthritis (103), being inversely related to the level of pathogenic effectors (such as IL-6 and TLR7) in human autoimmune rheumatic diseases (104). Furthermore, a recently described HERV LTR (HERVP71A, locus 6p22.1) was shown to serve as a tissue-specific enhancer for HLA-G gene expression in human extravillous trophoblasts, at the fetal-maternal interface, inhibiting the natural killer cell cytotoxicity and conferring immune tolerance to the developing placenta (106).

## HERVs and exogenous infections

HERVs have been proposed to have a role during exogenous viral infections, and such role could be either beneficial or harmful (16). It is worth to note that the interplay between exogenous and endogenous viruses is still not fully characterized, even if some evidences suggest that exogenous infection sustained by different viral species—among which HIV, herpesviruses and influenza—are able to modulate HERV expression (142–144). In the case of an upregulation of HERV expression, such cooperative action could increase the immune triggering exerted by HERV products and, especially in the presence of retroviral infections, possibly account for complementation of defective viruses and recombination events (16). Here, however, we will focus on the possible protective effects against exogenous infections exerted by HERV expression products, which can theoretically be able to restrict any step of the viral cycle (21).

**Figure 4:**
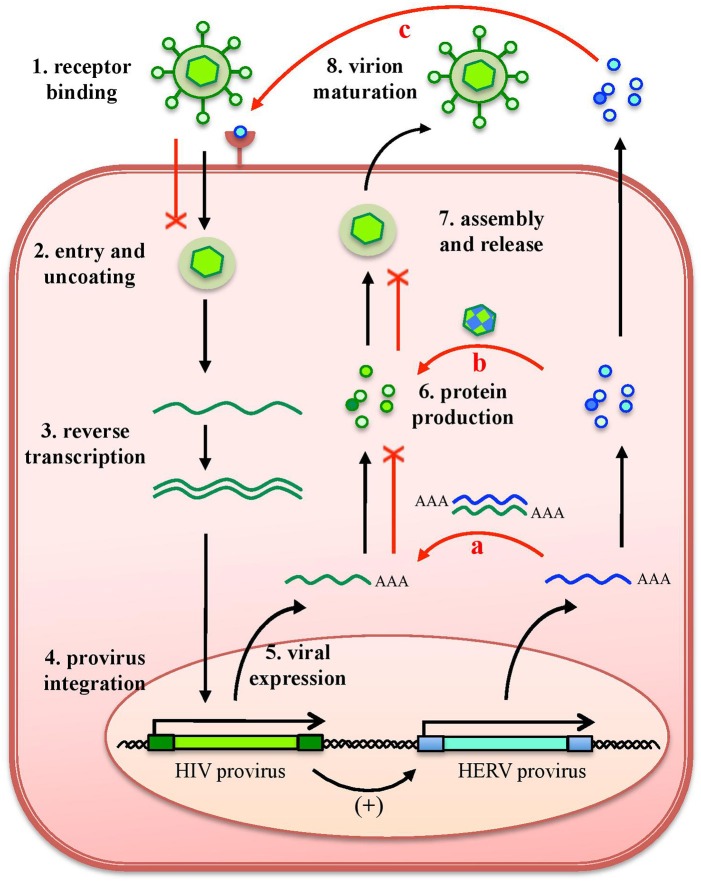
Protective effects exerted by HERVs against exogenous infections. The effects of HERV expression in the impairment of exogenous viruses (red lines) are represented in relation to HIV infection. The various steps of HIV replication are also schematized. Even if HERV expression could theoretically affect any of them, the major implications regard (a) the expression of HERV mRNAs, that can interact by complementarity with HIV RNA, could form dsRNA that is detected as a PAMP by cellular innate immunity sensors; (b) the complementation of HIV structural components with HERV proteins, possibly affecting HIV particles' assembly and release; and (c) the binding of HERV proteins or pseudoparticles to the same cellular receptor, preventing HIV binding and entry. Of note, these effects can be eventually sustained also by the possible upregulation of HERV expression by HIV infection.

As any organism or biological entity, viruses are subjected to an ensemble of selective pressures, being exerted by the host defenses as well as by the surrounding environment, including other microbes threatening the same host. Due to this, the different viral species evolved strategies to avoid the host antiviral systems and to compete with other viral populations, to assure their replication. In line with this, it is well known that a cell infected by a certain virus often become resistant to superinfection, developing a virus-induced viral resistance against members of the same species as well as different viruses (24). Considering HERVs, such cross-protection has been investigated mostly for exogenous retroviruses that share identity in their protein and nucleic acids, being more prone to interact with HERV products (16) (Figure 4). A partial resistance to exogenous infection could depend on the interference and blocking of the same cellular receptor by HERV-derived proteins or pseudo-viral particles (145, 146) (Figure 4). Inside the cell, the expression of HERV antisense transcripts has been proposed to confer protection against infections through the complementary interaction with homologous RNA sequences originated by exogenous retroviruses' expression, forming dsRNA molecules that can be recognized as PAMPs by human PRRs (26, 72) (Figure 4). Finally, in the case of HERV proteins' production, their identity with exogenous viral proteins could led to complementation events possibly affecting the formation of viral particles (Figure 4). Accordingly, a HERV-K(HML2) Gag protein was reported to co-assemble with HIV-1 Gag and to subsequently impair HIV-1 capsid formation as well as HIV-1 particles release and infectivity (147, 148) (Figure 4).

On the one side, all these direct interactions between HERV and exogenous viral products has been argued to had a role in the restriction and extinction of HERV-originating ancestral exogenous retroviruses (21). In line with this, Blanco-Melo and coauthors “resuscitated” two ancestral Env proteins: one was reconstructed from various HERV-T insertions in primate species, being more ancient, and the other was encoded by a human HERV-T provirus acquired about 20 million years later (149). The “older” Env was shown to bind the human receptor MTC1, and to be able to generate infectious pseudotyped MLV particles and syncytia (149). The “modern” HERV-T Env, albeit being still able to bind MTC1, lost these abilities and even downregulated MTC1 either at the cell surface (causing its internalization) or during secretion (blocking its transport), leading to its degradation (149). On the other side, the same interactions could have also contributed to the rapid evolution and adaptation of HERV genes, driving the selection of elements that could increase the range of restricted microbes and confer a sort of broad-viral protection (21). In this regard, HERV-K(HML2) Rec proteins upregulation has recently been reported during embryogenesis, leading to the specific stimulation of the IFN-induced viral restriction factor IFITM126 in epiblast and embryonic stem cells (56, 150). This finding led to the fascinating idea that Rec and the HML2 mRNAs, associated to it for nuclear export, might be detected in the cytosol, eliciting an innate anti-viral response thought to broadly inhibit embryonic viral infections (56).

## HERVs, cancer and anticancer strategies

In the context of cancer development, the ability of HERVs to modulating the immune system may have opposite effects, being potentially relevant to both the oncogenic processes and the anticancer defenses (23).

On the one side, HERV immunosuppressive functions might contribute to cancer progression by reducing the immune recognition and attack of tumor cells. As an example, the ISD peptide of an HERV-H Env (env60), namely H17, was shown to induce tumor cells' epithelial-to-mesenchymal transition, stimulating CCL19 chemokine expression and the subsequent recruitment and expansion of pluripotent immunoregulatory CD271^+^ cells (105). This mechanism was proposed to be critical for cancerous cell immune escape as well as for metastatic invasion and adhesion (105).

On the other side, the immunogenic properties of HERV expression combined with their general upregulation in cancer tissues, especially due to a broad epigenetic dysregulation, could hence represent an innovative therapeutic target (16). In fact, HERV products have been investigated in the field of anticancer immunotherapy, especially if expressed to higher extents (tumor-associated) or exclusively (tumor-specific) in transformed cells (16). As mentioned above, demethylating agents—commonly used in anticancer therapy—are known to induce a hypomethylated status liberating retrotransposon expression, which is the base of their therapeutic activity (25). In fact, such a stimulation led to the production of HERV bidirectional transcripts that can form dsRNA, sensed by cellular PRRs and directing thus a IFN-I and –III response against colorectal tumor cells (29). Accordingly, the individual knock-down of key immune players like MDA5, MAVS, and IRF7 in cancer cells significantly reduced the anticancer activity of DNA methyltransferase inhibitors (29). The activation of dsRNA sensors by HERV nucleic acids following the treatment with demethylating agents has been reported at the same time also by Chiappinelli et al. in ovarian cancer cells (27). Authors underlined that a major anticancer mechanism of demethylating agents is an induced IFN-I immune response mediated by the cytosolic dsRNA sensing of the multiple upregulated HERV transcripts (27). In particular, the cellular PRRs TLR3 and MDA5 have a pivotal role in such immune triggering, given that tumors showing high HERV expression present a concomitant significant activation of these viral sensors (27). Therefore, demethylating agents have been proposed either in association with active immunization therapies, to produce synergistic anticancer effects (151), or as a strategy to overcome primary resistance to immune checkpoint blocking therapies (25).

## Conclusions

A growing body of evidences suggests that the relationship between our genome and HERVs constitutes an intricate and multifaceted co-evolution spanning million of years throughout mammalian development. During such a long liaison, the detrimental effects exerted by HERVs have been balanced by beneficial activities that brought innovation and diversity to the human genome and physiology. Among these, the establishment of diverse HERV-mediated regulatory networks and the co-option of HERV proteins for pregnancy functions provided pivotal features characterizing our biological nature. Intriguingly, while HERVs are products of ancestral exogenous viral infections pervading primates, they became major contributors in shaping and improving the human antiviral immunity. Nowadays, they are still able to modulate it in an ambivalent way, suggesting that some new adaptive interplay between HERVs and our genome might still evolve *de novo* through sequence variation and fortuitous interactions (21). Additional studies are needed to characterize in detail the interaction between individual HERV products and specific immune receptors as well as the pathways involved in HERV-mediated modulation of innate responses. The characterization of such a complex interplay would allow to better understand the persistence of these long-time genomic residents and to finally clarify their role in human immune physiology and pathogenesis.

## Author contributions

NG and ET participated to the conception, drafting and revision of the manuscript and approved the final version.

### Conflict of interest statement

The authors declare that the research was conducted in the absence of any commercial or financial relationships that could be construed as a potential conflict of interest.
