# Supplementary material for: Human Endogenous Retroviruses Are Ancient Acquired Elements Still Shaping Innate Immune Responses
Source: Front Immunol. 2018 Sep 10;9:2039. doi: 10.3389/fimmu.2018.02039 (PMC6139349; doi:10.3389/fimmu.2018.02039)
Supplement: Supplementary file 1 [file Table_1.DOCX]

*Table S1. Alternative designations for the HERV groups and single members considered in the review in order of first appearance in the main text*

| *Name in the main text* | *Type of element* | *HERV group* | *locus* | *HUGO name* | *RepBase name* | *Other aliases* |
| --- | --- | --- | --- | --- | --- | --- |
| HERV-K(HML2) | group | - | - | ERVK | HERVK (int), LTR5 (LTR), LTR5Hs (LTR) | HERV-K, HML2 |
| HERV-K(HML10) | group | - | - | - | HERVKC4 (int), LTR14 (LTR) | HML10 |
| HERV-ADP | group | - | - | - | LTR71 (LTR) | HERVADP |
| HERV-FRD | group | - | - | ERVFRD | ERV3-1-I (int), LTR58 (LTR) | HERVFRD |
| ERVWE1 | locus | HERV-W | 7q21.2 | ERVW-1 | - | syncytin 1, enverin, envW, HERV- W-ENV, HERV-7q, HERV7Q |
| HERV-W | group | - | - | ERVW | HERV17 (int), LTR17 (LTR) | HERVW |
| ERVWE2 | locus | HERV-FRD | 6p24.1 | ERVFRD-1 | - | HERV-FRD, envFRD, ERVFRDE1, syncytin 2 |
| HERV-I | group | - | - | ERVI | HERV15 (int), LTR15 (LTR) | RHERVI, RRHERV-I, RTVL-I, Rtvli-int |
| HERV-H | group | - | - | ERVH | HERVH (int), LTR7 (LTR) | RTVL-H |
| HPAT5 | ncRNA | HERV-H | 6q27 | - | - | - |
| HERVKC4 | locus | HERV-K(HML10) | 6p21.33 | - | - | - |
| HERV-Fc1 | group | - | - | ERVFC1 | HERVFC (int), LTR46 (LTR) | - |
| env59 | protein | HERV-H | 2q24.1 | ERVH-10 | - | HERV-H3, envH3, envH/p59, env-59 |
| HERVP71A | LTR | HERV-P71 | 6p22.1 | - | HERVP71A-I (int), LTR71A (LTR) | - |
| HERV-T | group | - | - | ERVT | HERVS71 (int), LTR6 (LTR) | HERVT, S71, SSAV1, CRTK1, CRTK6, Hs5, HC2 |
| env60 | protein | HERV-H | 3q26 | ERVH-9 | - | envH2, HERV-H/env60, env-60 |

The HERV designation in the main text (column 1) as well as the correspondent aliases in HUGO HGNC (<https://www.genenames.org>), RepBase database (https://www.girinst.org/repbase/) and other eventual sources (literature, other databases) are reported.
